# SAT and SMT-Based Verification of Security Protocols Including Time Aspects †

**DOI:** 10.3390/s21093055

**Published:** 2021-04-27

**Authors:** Sabina Szymoniak, Olga Siedlecka-Lamch, Agnieszka M. Zbrzezny, Andrzej Zbrzezny, Miroslaw Kurkowski

**Affiliations:** 1 Department of Computer Science, Czestochowa University of Technology, Dabrowskiego 73, 42-200 Czestochowa, Poland; olga.siedlecka@icis.pcz.pl; 2 Faculty of Mathematics and Computer Science, University of Warmia and Mazury, Sloneczna 54, 10-710 Olsztyn, Poland; agnieszka.zbrzezny@matman.uwm.edu.pl; 3 Department of Mathematics and Computer Science, Jan Dlugosz University in Czestochowa, Armii Krajowej 13/15, 42-200 Czestochowa, Poland; a.zbrzezny@ujd.edu.pl; 4 Institute of Computer Science, Cardinal St. Wyszynski University, Woycickiego 1/3, 01-938 Warsaw, Poland; m.kurkowski@uksw.edu.pl

**Keywords:** security protocols, modelling, verification, sensor network protocols, time analysis

## Abstract

For many years various types of devices equipped with sensors have guaranteed proper work in a huge amount of machines and systems. For the proper operation of sensors, devices, and complex systems, we need secure communication. Security protocols (SP) in this case, guarantee the achievement of security goals. However, the design of SP is not an easy process. Sometimes SP cannot realise their security goals because of errors in their constructions and need to be investigated and verified in the case of their correctness. Now SP uses often time primitives due to the necessity of security dependence on the passing of time. In this work, we propose and investigate the SAT-and SMT-based formal verification methods of SP used in communication between devices equipped with sensors. For this, we use a formal model based on networks of communicating timed automata. Using this, we show how the security property of SP dedicated to the sensors world can be verified. In our work, we investigate such timed properties as delays in the network and lifetimes. The delay in the network is the lower time constraint related to sending the message. Lifetime is an upper constraint related to the validity of the timestamps generated for the transmitted messages.

## 1. Introduction

Contemporary fast life requires constant communication and information exchange. Modern communication takes place between people or institutions and increasingly between various types of devices equipped with sensors. Often, such devices are autonomous and equipped with software that uses artificial intelligence algorithms. There are many examples of such devices around us, ranging from cleaning robots to autonomous cars or even aircraft. Regardless of the type of participants, communication must remain secure, and the protocols used for this purpose should be verified and, if it is necessary, improved.

Security protocols (SP) are algorithms that ensure crucial security objectives in the communication process. Often for this purpose, cryptographic algorithms and techniques are applied. During communication, the most important data security goals are authorisation, information security and integrity, and cryptographic key distribution. For many years, many such protocols used timestamps. In addition, security protocols can be used in IoT systems [1,2,3,4,5]. It is necessary for the appropriate time-dependent management of cryptographic primitives (keys, passwords), especially to preserve and monitor the validity (lifetime) of the primitives.

Such a time-dependent management of primitives is sometimes crucial due to the increasing number of elements guaranteeing data confidentiality, such as encryption keys, timestamps, and number of protocol steps. Having precisely defined lifetimes of primitives, administrators have full control over the data necessary to maintain secure communication and can choose parameters for it. In the case of protocols used for devices equipped with sensors, such tasks are even more complicated and the time aspect even more important. These devices usually have limited resources, relatively small memory, limited battery life, hence the limited energy, and do not have the computing power to generate complex ciphertexts. Therefore, it is essential to be able to match the protocol and its parameters to such limitations.

Since the first projects and implementations, many SPs have been developed and successfully used. Unfortunately, many examples have shown that protocols sometimes can be cheated by malicious intruders [6,7]. That is the reason for investigating SP properties, especially widely understood correctness. From the 1990s, many well-grounded methodologies and practically used verification tools helped in verifying and designing SP. The first attempts of checking SP properties were by testing real or virtual environments. Nevertheless, in this case, we must say that even many testing days, months, or years cannot answer the question of whether the investigated SP achieves its goal or not. After a consierable period of testing, we can only say that the system works correctly so far.

Another way of SP property verification is by using the formal deduction methods. Logics created for reasoning about SP properties were widely known as authentication logics. These logics have been successfully applied to investigating SP correctness [8]. Formal reasoning allowed to discover errors in several SP schemes by proving the bugs or weaknesses in their constructions. However, such methods had serious problems. Imperfect deductive systems or the huge size of proof trees caused the verification process to be ineffective. In cases of logics with well-grounded (adequate or complete) semantics, it could be seen that inference can be made on the semantic side by examining the computational model on which the semantics of logic was based. This was one of the reasons why authentication logics gradually ceased to be popular. In fact, it can even be said that in this field, authentication logics have been successfully supplanted by model verification methods that examine computational structures representing the protocol’s executions.

Taking the above into account, we should state that the most important formal ways for the SP properties’ verification are, from many years, model checking techniques. In such approaches, an appropriate and adequate formal model of the protocol executions is created and investigated. There were many concepts of SPs’ modelling and many algorithms of how created formal models can be searched. In the case of SP verification, we have to notice a few beneficial and very well-grounded approaches. The first one is the well-known AVISPA tool [9], which uses HLPSL, a specially designed language for SP specification. Others are Scyther proposed by Cremers and Mauw [10], and ProVerif by Blanchet [11]. These tools allow for automatic investigations of SP properties, especially their correctness. We can also annotate Kurkowski’s solutions in the VerICS tool, where protocols’ executions were modelled as work of networks of synchronised automata [12]. Such methods and tools are still developed and practically used to verify many protocols. See for example [13,14,15,16,17,18].

Here, we have to annotate that only several solutions that can express or investigate SP time properties have been introduced so far. It is a very important problem because for several years developers added into protocols schemes time tickets and their lifetimes’ values. It should be noted that time modelling is not an easy problem at all. In this case several ways of expressing time and time properties in formal computer systems models were introduced [14,16,19,20,21].

Our work follows for works of Jakubowska and Penczek [22], and Kurkowski and Penczek [12]. In the first approach, the authors did not go beyond investigating security properties connected with only one SP’s session. In [23] new formal, discrete, mathematical methods for protocol verification have been introduced. With the presented methods, it is possible to prove the correct operation of the time-dependent security protocol. This model was used to study authentication processes. In further works, one can find the next considerations regarding network delays and calculations regarding the duration of the communication session. The analysed time constraints revealed the influence of time on protocol security.

Tne above-mentioned allow further successful investigations that were published in [24,25,26,27]. In the research conducted so far, we have used synchronised automata networks, modelling by chains of states, SAT, and SMT techniques. The latest research concerns the temporal aspects. In this paper, we present developed methods and the newest improvements and the extension to further examples of protocols. The developed model showed the strengths and weaknesses of the tested protocols and the fact that one can use even potentially weak protocols with appropriate time constraints, which can be particularly useful in the case of sensor systems. It can also be noticed that there is a way to increase the protocol’s security by strengthening key points. We used the tools we implemented as well as SAT and SMT solvers for the experimental research.

In our work we distinguish two types of intruder behaviour. The first one is the actual attack where the intruder can get confidential data or cheat someone their identity. We can precisely say that such behaviours are attacks. The second one is some case of Man in the Middle situation, where the intruder, staying between two communicating sides, can only receive and resend the whole message during protocols execution without breaking ciphertexts, possessing secret data, or cheat someone their identity. The second type of behaviour we call Man in the Middle Intruder’s behaviour (MiTM behaviour). Such a behaviour is a simple passive resending of messages. In some papers, such behaviours are called attacks, which may be confusing in understanding the problem.

The main contributions of the paper are as follows:

- Formal modelling of timed security protocols dedicated to sensor devices on the example of the SNEP protocol,
- Timed analyses of the SNEP protocol,
- Applying SAT- and SMT-based to reachability testing,
- And analyses of dependence of lifetime on delays.

### Related Work

In the last several years only a few research groups have investigated problems connected to modern timed solutions in SP schemes. In works [28] the authors developed and considered THLPSL language (Timed High-Level Protocols Specification Language). However, only the simplest timed properties were investigated. These works did not contain considerations about networks’ delays, times of messages compositions, and ciphering/deciphering. Szymoniak et al. [25,29,30,31] and Li et al. [32] took into account networks delays. It allowed consideration about time dependencies between the possibility of protocol executing and lifetimes of time tickets values. Li et al. created an automatic system for computing proper time dependencies between network delays and lifetimes. However, they only considered one value each for lifetime and delay. In the case of sensors devices devoted protocols, only a few papers were dedicated to verifying their properties. Here the AVISPA tool was used. However, only untimed versions of such protocols were taken into account [33].

The rest of the paper is organised as follows. In the next chapter, we describe example protocols that will help us explain how to model and verify. Next, we present the necessary definitions for timed automata and the network of timed automata. In a further chapter, we present the formal language and computational mathematical structure. In the next section, we will describe how to apply SAT and SMT techniques. Then we present the research assumptions and the results of the experiments. We will present the conclusions in the last section.

Table 1 presents the notations used in the article together with explanations:

## 2. Sample Protocols

In this section, we will present two examples of protocols. The first one is the Woo Lam Pi standard security protocol used in more complex communication protocols. The second one—the SNEP protocol is part of sensor communication systems. Both will be used later in work to explain formal modelling and present the experimental results.

### 2.1. WooLamPi Protocol

We have chosen the WooLamPi (WLP) protocol for our presentation because, like SNEP, it uses symmetric cryptography and has similar construction of some transmitted data (with double encryption). The WLP protocol was designed by Thomas Y. C. Woo and Simon S. Lam’s and described in work [34]. This protocol uses symmetric cryptography, which is a one-way authenticator using a trusted server. WLP is the basis for the next version of this protocol (WooLamPi1, WooLamPi2, and WooLamPi3). In the original, the WooLamPi protocol are used nonces (pseudorandom numbers).

To perform research with time parameters, we, in a usual way, change nonce values into timestamps, thus gaining the opportunity to take into account time and be able to consider and verify time properties. It is important to note that changing nonces to timestamps is a practice confirmed by suitable security standards and widely used in real solutions in this area. Nonces and timestamps as time-variant parameters are officially considered to be equivalent from a time-dependent properties point of view. To increase the security level of communication, we can add pseudorandom values (nonces) in two ways: Firstly as part of the timestamp or second as an additional part of the whole message. The details of such a mechanism can be found in the ISO/IEC 9798 norm.

The timed version of this protocol in the so-called ’Alice and Bob’ notation is as follows:
$\alpha_{1}\;\;$A→B:$I_{A}$$\alpha_{2}\;\;$B→A:$T_{B}$$\alpha_{3}\;\;$A→B:${\langle T_{B}\rangle }_{K_{AS}}$$\alpha_{4}\;\;$B→S:${\langle I_{A}\cdot {\langle T_{B}\rangle }_{K_{AS}}\rangle }_{K_{BS}}$$\alpha_{5}\;\;$S→B:${\langle T_{B}\rangle }_{K_{BS}}.$

The WLP protocol consists of five steps. In the first step, Alice (user *A*) sends its identifier to Bob (user *B*), informing him of her willingness to initiate a new session. In response, Bob generates a timestamp $T_{B}$ and sends it to Alice. Then Alice creates a message in which the timestamp $T_{B}$ is received. Bob forwards this message to the server (marked with the letter *S*) by adding the $I_{A}$ identifier to it. The entire message is encrypted with a symmetric key $K_{BS}$ shared between *B* and the server *S*. In the last step of this protocol, the server sends its timestamp to Bob in the message encrypted with the $K_{BS}$ symmetric key.

The WLP protocol has many versions; their descriptions can be found in [34]. Each version of the WLP has the same number of steps, and the same operations are performed in the first and second steps. Let us show the next version from the third step, where some modifications were made to ensure the security of the protocol:
$\alpha_{3}\;\;$A→B:${\langle I_{A}\cdot I_{B}\cdot T_{B}\rangle }_{K_{AS}}$$\alpha_{4}\;\;$B→S:${\langle I_{A}\cdot I_{B}\cdot {\langle I_{A}\cdot I_{B}\cdot T_{B}\rangle }_{K_{AS}}\rangle }_{K_{BS}}$$\alpha_{5}\;\;$S→B:${\langle I_{A}\cdot I_{B}\cdot T_{B}\rangle }_{K_{BS}}.$

The message from the third step includes the generated timestamp $T_{B}$ and the identifiers of both users. The following two messages also contain the identifiers of both users.

For the WooLamPi2 protocol, the steps are as follows:
$\alpha_{3}\;\;$A→B:${\langle I_{A}\cdot T_{B}\rangle }_{K_{AS}}$$\alpha_{4}\;\;$B→S:${\langle I_{A}\cdot {\langle I_{A}\cdot T_{B}\rangle }_{K_{AS}}\rangle }_{K_{BS}}$$\alpha_{5}\;\;$S→B:${\langle I_{A}\cdot T_{B}\rangle }_{K_{BS}}.$

From step 3, the initiator ID (user *A*) has been added for all messages.

For the WooLamPi3 protocol, the base protocol is as follows:
$\alpha_{4}\;\;$B→S:${\langle I_{A}\cdot {\langle T_{B}\rangle }_{K_{AS}}\rangle }_{K_{BS}}$$\alpha_{5}\;\;$S→B:${\langle I_{A}\cdot T_{B}\rangle }_{K_{BS}}.$

There are no severe changes in the last steps. In the fourth and fifth steps, the initiator’s ID appears. No attack on secrecy or authentication was detected on any of the WooLamPi protocols. Only Man in the Middle behaviour is possible (without taking over confidential information).

### 2.2. SNEP Protocol

The Secure Network Encryption Protocol (SNEP) is a security protocol designed for sensor networks. It was introduced at [35] by Perrig et al. This protocol ensures data confidentiality, two-way data authentication, and its validity. SNEP provides semantic security in the form of a strong security feature. Thanks to this, the intruder cannot infer the content of the plaintext from the multiple-encrypted message.

In the SNEP protocol, authors have introduced an additional cryptographic mechanism to reduce the energy required to transmit random data over the wireless channel. This mechanism uses two counters (one for each communication direction). The communicating parties share these counters. However, they are not sent and incremented with each message as in the traditional approach. The exchange of counters takes place using an additional protocol (counter exchange protocol) that synchronises the counters between the communicating parties. In turn, the Message Authentication Code (MAC) achieves two-way authentication and data integrity.

SNEP uses independent symmetric encryption keys and MAC codes for all its operations. Here symmetric encryption works in a counter mode of operation, and MAC function over data is a function that computes a MAC code of data with additional parameters *K*-key and *C*-counter. Firstly we assume that the two communicating parties *A* and *B* share an $X_{AB}$ master secret key and obtain from it independent encryption keys for each communication direction and MAC keys using some fixed pseudorandom function *F* over $X_{AB}$. In the further part of the analysis, we will use the keys derived from the key $X_{AB}$, namely the keys $K_{AB}$ and $K_{BA}$. They are symmetric keys, known by both users *A* and *B*, for communication between them. It should be emphasised that they are not mathematically the same keys, although they perform the same data encryption role. This is essential information because of modelling details in the following sections of the paper.

Encrypted data in the SNEP protocol takes the following format:

(1) $$E={\langle D\rangle }_{K,C}$$

where ${\langle D\rangle }_{K,C}$ denotes a ciphertext that contains plaintext *D* encrypted in a counter mode of operation with the symmetric encryption key and the counter *C*.

Observe that ciphertexts encrypted with keys $K_{AB}$, $K_{BA}$, and the counter *C*: ${\langle D\rangle }_{K_{AB},C}$, and ${\langle D\rangle }_{K_{BA},C}$ are not identical.

We will describe the MAC message authentication code of the data *E* computed with the parameters *K* (the key) and *C* (the counter) as ${\langle E\rangle }_{M(K,C)}$.

The SNEP protocol original (untimed) version consists of two parts. The goal of the first one is setting the user’s counters, and the second is the part responsible for authentication.

Let us describe the first part as follows:
$\alpha_{1}\;\;$A→B:$C_{A}$$\alpha_{2}\;\;$B→A:$C_{B}\cdot {\langle C_{B}\rangle }_{M(K_{BA}^{\prime },C_{A})}$$\alpha_{3}\;\;$A→B:${\langle C_{B}\rangle }_{M(K_{AB}^{\prime },C_{A})}.$

In the first step the user *A* send to the user *B* the counter value $C_{A}$. In the second step, the user *B* sends to *A* its counter value $C_{B}$ with this value’s MAC code computed using the key $K_{BA}^{\prime }$ and obtained $C_{A}$ value. Observe that both users *A* and *B* know $X_{AB}$ and can compute from keys $K_{BA}^{\prime }$ and $K_{AB}^{\prime }$. In the third step *A* send to *B* the MAC code of the value $C_{B}$ computed using the key $K_{AB}^{\prime }$ and value of its counter $C_{A}$. As we mentioned before, in this part of SNEP, *A* and *B* exchange their counter values. This part can be done rarely in the usual communication process, for example, once a day.

The second part of SNEP is devoted to one side authentication of the user *B* to the user *A*. This part should be done as a start in every communication session. This part consists of two steps:
$\alpha_{4}\;\;$A→B:$N_{A}\cdot R_{A}$$\alpha_{5}\;\;$B→A:${\langle R_{B}\rangle }_{K_{BA},C_{B}}\cdot {\langle {\langle R_{B}\rangle }_{K_{BA},C_{B}}\rangle }_{M(K_{BA}^{\prime },N_{A}\cdot C_{B})}.$

In the $\alpha_{4}$ step, the user *A* generates its own nonce $N_{A}$ and sends it to Bob with a special code or text $R_{A}$ that plays the role of the request for the answer. Observe that $N_{A}$ is generated strictly at the moment of starting this part of SNEP. Before this time, the number $N_{A}$ did not exist. In the $\alpha_{5}$ step, *B* answers to *A* through a message containing the ciphertext ${\langle R_{B}\rangle }_{K_{BA},C_{B}}$ (remember that only *A* and *B* know the key). This message also contains the MAC computation value of ${\langle R_{B}\rangle }_{K_{BA},C_{B}}$ computed with using the $N_{A}$ value, freshly generated and obtained by *B* in $\alpha_{4}$ step ($R_{B}$, similarly $R_{A}$ is a text or a code). After the MAC code has been appropriately verified, *A* will know that *B* generated a response to the message request. The usage of $N_{A}$ ensures the high freshness of the response. As we mentioned before, this part of SNEP guarantees a one-side authentication of the user *B* to the user *A*. Remember additionally that only *A* and *B* knows $X_{AB}$ and computed from this keys $K_{AB},K_{BA},K_{AB}^{\prime }$, and $K_{BA}^{\prime }$. This SNEP’s part can be executed in the reverse direction and confirm the authentication of *A* to *B*.

For the next consideration point of view, we should note that the authors of the SNEP protocol use the MAC function because of the small computational and memory power of sensors devices. Today on the cryptographic market, there are many examples of light cyphers, for example, Cha-Cha or Salsa, that can be successfully used in investigated devices (see [36,37,38]). Thus in the following considerations, we use a slightly modified version of SNEP, with symmetric light encryption instead of the MAC function. From the cryptographic point of view, there is no difference here. Additionally, we add for the SNEP scheme timestamps that allow investigation about SNEP time properties.

In our research, we model and check the security time properties of two versions of SNEP. In the first one, both parts of the protocol (reconciliation of meters and proper authorisation) follow each other. In this case, the protocol scheme is as follows:
$\alpha_{1}\;\;$A→B:$T_{A}\cdot C_{A}$$\alpha_{2}\;\;$B→A:$T_{B}\cdot C_{B}\cdot {\langle T_{A}\cdot C_{A}\cdot T_{B}\cdot R_{B}\rangle }_{K_{BA}^{\prime }}$$\alpha_{3}\;\;$A→B:$N_{A}\cdot R_{A}\cdot {\langle T_{A}\cdot C_{A}\cdot T_{B}\cdot R_{B}\rangle }_{K_{AB}^{\prime }}$$\alpha_{4}\;\;$B→A:${\langle T_{B}\cdot R_{B}\cdot C_{B}\rangle }_{K_{BA}}\cdot {\langle N_{A}\cdot T_{B}\cdot R_{B}\cdot {\langle C_{B}\cdot T_{B}\cdot R_{B}\rangle }_{K_{BA}}\rangle }_{K_{BA}^{\prime }}$.

If we allow a certain distance in time and re-establish communication between users, we can write SNEP in this way:
$\alpha_{1}\;\;$A→B:$T_{A}\cdot C_{A}$$\alpha_{2}\;\;$B→A:$T_{B}\cdot C_{B}\cdot {\langle T_{A}\cdot C_{A}\cdot T_{B}\cdot R_{B}\rangle }_{K_{BA}^{\prime }}$$\alpha_{3}\;\;$A→B:${\langle T_{A}\cdot C_{A}\cdot T_{B}\cdot R_{B}\rangle }_{K_{AB}^{\prime }}$$\alpha_{4}\;\;$B→A:$I_{B}\cdot I_{A}$$\alpha_{5}\;\;$A→B:$N_{A}\cdot R_{A}$$\alpha_{6}\;\;$B→A:${\langle T_{B}\cdot R_{B}\cdot C_{B}\rangle }_{K_{BA}}\cdot {\langle N_{A}\cdot T_{B}\cdot R_{B}\cdot {\langle C_{B}\cdot T_{B}\cdot R_{B}\rangle }_{K_{BA}}\rangle }_{K_{BA}^{\prime }}$.

Both proposed versions meet all assumptions of the original version of SNEP.

For clarity of further analysis, let us also show the MiTM behaviour for one SNEP version. Let us present it on a shorter version of the protocol. For such a situation to occur, we need to interlace the two executions. In the first execution ($\alpha_{i}^{1}$), the intruder impersonates user B and communicates with A. In the second execution ($\alpha_{i}^{2}$), they impersonate A and communicates with B.
$\alpha_{1}^{1}\;\;$A→I(B):$T_{A}\cdot C_{A}$$\alpha_{1}^{2}\;\;$I(A)→B:$T_{A}\cdot C_{A}$$\alpha_{2}^{2}\;\;$B→I(A):$T_{B}\cdot C_{B}\cdot {\langle T_{A}\cdot C_{A}\cdot T_{B}\cdot R_{B}\rangle }_{K_{BA}^{\prime }}$$\alpha_{2}^{1}\;\;$I(B)→A:$T_{B}\cdot C_{B}\cdot {\langle T_{A}\cdot C_{A}\cdot T_{B}\cdot R_{B}\rangle }_{K_{BA}^{\prime }}$$\alpha_{3}^{1}\;\;$A→I(B):$N_{A}\cdot R_{A}\cdot {\langle T_{A}\cdot C_{A}\cdot T_{B}\cdot R_{B}\rangle }_{K_{AB}^{\prime }}$$\alpha_{3}^{2}\;\;$I(A)→B:$N_{A}\cdot R_{A}\cdot {\langle T_{A}\cdot C_{A}\cdot T_{B}\cdot R_{B}\rangle }_{K_{AB}^{\prime }}$$\alpha_{4}^{2}\;\;$B→I(A):${\langle T_{B}\cdot R_{B}\cdot C_{B}\rangle }_{K_{BA}}\cdot {\langle N_{A}\cdot T_{B}\cdot R_{B}\cdot {\langle C_{B}\cdot T_{B}\cdot R_{B}\rangle }_{K_{BA}}\rangle }_{K_{BA}^{\prime }}$$\alpha_{4}^{1}\;\;$I(B)→A:${\langle T_{B}\cdot R_{B}\cdot C_{B}\rangle }_{K_{BA}}\cdot {\langle N_{A}\cdot T_{B}\cdot R_{B}\cdot {\langle C_{B}\cdot T_{B}\cdot R_{B}\rangle }_{K_{BA}}\rangle }_{K_{BA}^{\prime }}$.

For both versions of the SNEP protocol (as presented earlier WooLamPi), the intruder’s presence inside the communication is not a direct attack. The intruder does not gain confidential information and can only store entire ciphertexts. It should be emphasised that this is also an undesirable situation that we wish to avoid.

## 3. Networks of Synchronised Timed Automata

Now we start introducing formal structures used for protocols’ modelling and verification. As mentioned before, we model executions of timed security protocols as a network of synchronised timed automata. Our work bases on methodology introduced precisely in [12]. Below we show needed formal definitions due to such networks required for the next considerations conducted in the following sections. As an example, we will show how to model the executions of a protocol by the runs of a network of synchronised timed automata, where each timed automaton represents one component of the protocol. We will also introduce automata representing messages transmitted during a protocol execution and automata representing users’ knowledge. We need to model the executions of the protocol according to the knowledge acquired by users. We have introduced an appropriate synchronisation between the automata of the network.

Let $X=\{x_{0},\ldots ,x_{n-1}\}$ be a finite set of variables, called clocks. By clock valuation we mean a total function v:X↦IR that assigns to each clock *x* a non-negative real number v(x). We also denote by ${I\;R}^{|X|}$ the set of all the clock valuations.

Let x∈X, c∈IN, and ∼∈{<,⩽,=,⩾,>}. Clock constraints over X are conjunctions with information about comparisons of a clock with a time constant from the set of non-negative natural numbers IN. The set C of clock constraints over the set of clocks X is defined by the following grammar:

cc:=x∼c|cc∧cc.

Additionally let *v* be a clock valuation, and cc∈C. We say that a clock valuation *v* satisfies a clock constraint cc, iff cc evaluates to true using the clock.

**Definition** **1.** *A timed automaton [19,39] (TA, for short) is a seven-tuple $A=(Act,L,l{}^{0},X,AP,$E,V), where:*

- *Act is a finite set of* actions,
- *L is a finite set of* locations,
- *$l{}^{0}\in L$ is the* initial location,
- *X is a finite set of clocks,*
- *AP is a set of atomic propositions,*
- *$E\subseteq L\times Act\times C\times 2^{X}\times L$ is a* transition relation,
- *$V:L\mapsto 2^{AP}$ is a valuation function assigning to each location a set of atomic propositions.*

*Every element e from the set E is denoted by $l\overset{a,\mathrm{cc},X}{\frac{\;}{\;\;\;}\;\;\;⟶}l^{\prime }$. It represents a transition from the location l to the location $l^{\prime }$, executing the action a, with the set X⊆X of clocks to be reset, and with the clock condition cc∈C defining the enabling condition (guard) for e.*

The clocks of a timed automaton allow for expressing the timing properties. A guard restricts the execution of the transition and at the same time does not force the execution of this transition.

We give at the next sections several examples of timed automata used for modelling of protocols executions.

### 3.1. Semantics of Timed Automata

Let $A=(Act,L,l{}^{0},E,X)$ be a timed automaton. A concrete state of A is defined as an ordered pair (l,v), where l∈L and $v\in {I\;R}_{+}^{|X|}$ is a clock valuation (${I\;R}_{+}$ denotes the set of positive real numbers). In what follows, by [[cc]] we mean a set of all the valuations *v* that satisfy a time condition cc. By v[X:=0] we mean the valuation $v^{\prime }$ such that it assigns 0 to all clocks from the set *X* and it agrees with *v* on all the remaining clocks, i.e., $v^{\prime }\left(X\right)=\left\{0\right\}$ and $v^{\prime }\left(x\right)=v\left(x\right)$ for each x∈X∖X. The formal definitions of these notations can be found in [12].

The *state space* of A is a transition system $C\left(A\right)=\left(Q,s{}^{0},\overset{}{\to },V\right)$ [39], where:

- $Act\cup {I\;R}_{+}$ is the set of labels,
- $Q=L\times {I\;R}_{+}^{|X|}$ is the set of all the *concrete states*,
- $s{}^{0}=\left(l{}^{0},v{}^{0}\right)$ with $v{}^{0}\left(x\right)=0$ for all x∈X is the *initial state*,
- $\overset{}{\to }\;\subseteq Q\times \left(Act\cup {I\;R}_{+}\right)\times Q$ is the *transition relation*, defined by action- and time successors as follows:
  – For any $\delta \in {I\;R}_{+}$, $\left(l,v\right)\overset{\delta }{\to }\left(l,v+\delta \right)$ (*time successor*),
  – For a∈Act, $\left(l,v\right)\overset{a}{\to }\left(l^{\prime },v^{\prime }\right)$ iff (∃cc∈C)(∃X⊆X) such that $l\overset{a,\mathrm{cc},X}{\frac{\;}{\;\;\;}\;\;\;⟶}l^{\prime }\in E$, v∈[[cc]] and $v^{\prime }=v\left[X:=0\right]$ (*action successor*).
- A valuation function $V:Q\mapsto 2^{AP}$ is defined such that V((l,v))=V(l) for all (l,v)∈Q.

Intuitively, a time successor does not change the location *l* of a concrete state, but it increases the clocks. An action successor corresponding to an action *a* is executed when the guard cc holds for *v* and the valuation $v^{\prime }$ obtained by resetting the clocks in *X*.

We shall say that a location *l* is reachable in a given transition system for a timed automaton A if for some clock valuation *v* the state (l,v) is reachable in this transition system.

For (l,v)∈Q and $\delta \in {I\;R}_{+}$, let (l,v)+δ denote (l,v+δ). A *$s_{0}$-run*ρ of A is a maximal sequence $\rho =s_{0}\overset{\delta_{0}}{\to }s_{0}+\delta_{0}\overset{a_{0}}{\to }s_{1}\overset{\delta_{1}}{\to }s_{1}+\delta_{1}\overset{a_{1}}{\to }s_{2}\overset{\delta_{2}}{\to }\ldots ,$ where $a_{i}\in Act$ and $\delta_{i}\in {I\;R}_{+}$, for each i∈IN.

Now, we are going to use networks of timed automata (NTA) for modelling executions of the protocol and the knowledge of the participants.

### 3.2. Product of a Network of Timed Automata

Now we consider a product **A** of i-th timed automata constructed before. In a standard way, **A** is a timed automaton too, so it consists of seven components. A state of **A** is a i-tuple of states of constituents automata. In **A**, a transition labelled by α-label can be executed iff in all constituents automata that possess α, at least one transition labelled by α can be executed too. All other conditions that determine other components of **A** are intuitively similar (see [12] for detail).

## 4. Formal Language and Computational Structure

This section focuses on the essential elements of the formal language necessary to build a computational structure. The entire formal model and the computational structure was shown in [31]. Initially, a protocol step definition is required.

In the following considerations, among these introduced before, we use the additional notations mentioned in Table 2.

Formally a timed protocol step (including delay in the network) is defined by two tuples, where:
$\alpha^{1}\;\;$=$\left(S_{\to },R_{\leftarrow },L\right)$$\alpha^{2}\;\;$=(τ,D,X,G,tc).

In this notation, *L* is the message sent in the step, $S_{\to }$ is the sender, and $R_{\leftarrow }$ is a receiver. From studying the properties of temporal security protocols point of view, the second tuple is very important. Here, τ is the time of sending the message, *D* is a delay in the network, *X* is the set of letters necessary to compose the message *L*, *G* is the set of letters that the sender must generate to compose the message *L*, and tc is the set of timed conditions that should be met in order to enable the protocol execution.

As an example, we presented the formal definition of the WLP Protocol:

$$\alpha_{1}\;=\;\left(\alpha_{1}^{1},\alpha_{1}^{2}\right)\;\;\;\alpha_{1}^{1}\;=\;\left(A,B,I_{A}\right)$$

$$\alpha_{1}^{2}\;=\;\left(\tau_{1},D_{1},\left\{I_{A}\right\},\left\{\emptyset \right\},true\right)$$

$$\alpha_{2}\;=\;\left(\alpha_{2}^{1},\alpha_{2}^{2}\right)\;\;\;\alpha_{2}^{1}\;=\;\left(B,A,\tau_{B}\right)$$

$$\alpha_{2}^{2}\;=\;\left(\tau_{2},D_{2},\left\{\tau_{B}\right\},\left\{\tau_{B}\right\},true\right)$$

$$\alpha_{3}\;=\;\left(\alpha_{3}^{1},\alpha_{3}^{2}\right)\;\;\;\alpha_{3}^{1}\;=\;\left(A,B,{\langle \tau_{B}\rangle }_{K_{AS}}\right)$$

$$\alpha_{3}^{2}\;=\;\left(\tau_{3},D_{3},\left\{\tau_{B},K_{AS}\right\},\left\{\emptyset \right\},\tau_{3}+D_{3}-\tau_{B}\leq L_{\tau_{B}}\right)$$

$$\alpha_{4}\;=\;\left(\alpha_{4}^{1},\alpha_{4}^{2}\right),\;\;\;\alpha_{4}^{1}\;=\;\left(B,S,{\langle I_{A},{\langle \tau_{B}\rangle }_{K_{AS}}\rangle }_{K_{BS}}\right),$$

$$\alpha_{4}^{2}\;=\;\left(\tau_{4},D_{4},{\{I_{A},{\langle \tau_{B}\rangle }_{K_{AS}}\rangle }_{K_{BS}}\right\},\left\{\emptyset \right\},\tau_{4}+D_{4}-\tau_{B}\leq L_{\tau_{B}}).$$

$$\alpha_{5}\;=\;\left(\alpha_{5}^{1},\alpha_{5}^{2}\right),\;\;\;\alpha_{5}^{1}\;=\;(S,B,{\langle \tau_{B}\rangle }_{K_{AS}}),$$

$$\alpha_{5}^{2}\;=\;(\tau_{3},D_{5},\left\{\tau_{B},K_{BS}\right\},\left\{\emptyset \right\},\tau_{5}+D_{5}-\tau_{B}\leq L_{\tau_{B}}).$$

According to the WLP protocol structure, we will discuss the third step in detail. At this point, user *A* sent to user *B* a message. To compose this message, user *A* needs the following set of objects: $\left\{\tau_{B},K_{AS}\right\}$. The set of generated objects is empty. It means that user *A* does not need to generate any objects in this step. The sending time of this message was extended by delay in the network and shortened by the timestamp value. This time must be less than or equal to the assumed lifetime value. The previous and next steps of the WLP protocol should be considered in the same way.

We can model executions of security protocols as specially designed discrete, mathematical transition structures. One of these structures is a network of synchronised timed automata. Our network works according to the formal definition of a network of synchronised timed automata presented in [12,21]. In this network, the global state is the tuple that consists of precisely one state from each automaton. The initial state of the network is a tuple that consists of all the initial states of all automata. A given action α can be executed in the network if and only if α is enabled in all the automata in which this action appears.

We consider two types of timed automata in our network: Knowledge and execution automata. Execution automata model executions protocol steps together with time conditions. The second type of automata models the process of gaining knowledge by users. These automata are synchronised by labels that allow modelling the need to acquire specific knowledge by users to execute the next protocol step.

**Figure 1:**
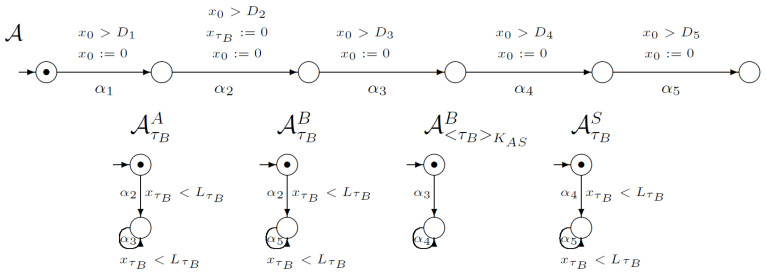
Network of synchronised timed automata for the WooLamPi protocol.

Figure 1 shows a part of the network of synchronised timed automata. The network models an execution of the WooLamPi protocol (including delays in the network). In this picture, we marked the initial global state of our network, which is a tuple of the local initial states (denoted by the black dot). Automaton A models the execution of all protocol steps and time conditions. Each of A’s transitions are connected with the proper protocol’s step. The automata in Figure 1 model the changes in users’ knowledge during the execution of the protocol. For example, the first automaton $A_{\tau_{B}}^{A}$ models gaining knowledge about the timestamp $\tau_{B}$ by the user *A*. The first, initial state of $A_{\tau_{B}}^{A}$ models a state where the user *A* does not possess knowledge about $\tau_{B}$. The first transition in $A_{\tau_{B}}^{A}$ labelled by $\alpha_{2}$ is synchronised with the second transition of execution automaton A. Such synchronisation guarantees possessing knowledge about the timestamp $\tau_{B}$ by the user *A*. The second local state of $A_{\tau_{B}}^{A}$ describes a situation when the user *A* knows the timestamp $\tau_{B}$. A loop labelled with the label $\alpha_{3}$ is connected with the situation when the knowledge about $\tau_{B}$ is necessary to execute another step connected with transitions in execution automaton A, when the user *A* needs the knowledge about the timestamp $\tau_{B}$. Such ideas and constructions were proposed in [12]. For a network of synchronised timed automata, we use the global clock $x_{0}$ and clocks $x_{\tau_{U}}$ for all timestamps created by the users. For the presented part of the network, we use the clock $x_{\tau_{B}}$ that measures the time from the point of creating the timestamp $\tau_{B}$. Please note that after each transition we reset a global clock $x_{0}$ and compare its value with the appropriated value of *D*, according to protocol structure. In addition, in the case of knowledge automata, we compare the value of clock $x_{\tau_{B}}$ with $L_{1}$ value, according to the protocol structure.

Note that each transition compares the global clock $x_{0}$ with the appropriated delay *D*, and then resets $x_{0}$. In the case of knowledge automata, the value of clock $x_{\tau_{B}}$ is compared with the lifetime $L_{\tau_{B}}$, according to the protocol structure.

**Figure 2:**
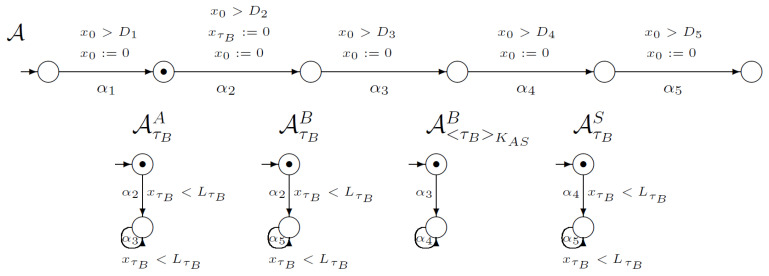
The first step of the WooLamPi protocol.

The initial global state changes to another by executing the action $\alpha_{1}$—the only one enabled in the initial state. The result of executing the action $\alpha_{1}$ is shown in Figure 2. Observe that according to protocol execution, the users’ knowledge is not changed in the first step.

**Figure 3:**
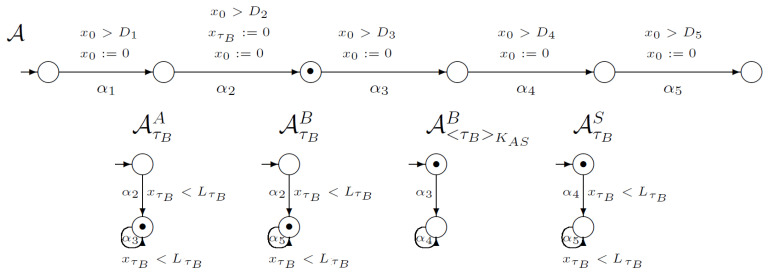
The second step of WooLamPi protocol.

After that, the second step of the protocol can be executed (Figure 3). The second transition in the automaton *A* is synchronised with the first transitions in automata $A_{\tau_{B}}^{A}$ and $A_{\tau_{B}}^{B}$. For the WLP protocol, the second step’s execution requires changing user *B*’s knowledge about its timestamp because user *B* generates timestamp $\tau_{B}$. In addition, user *A*’s knowledge about this ticket is changing. User *A* possess this timestamp. Clock $x_{\tau_{B}}$ must be reset.

**Figure 4:**
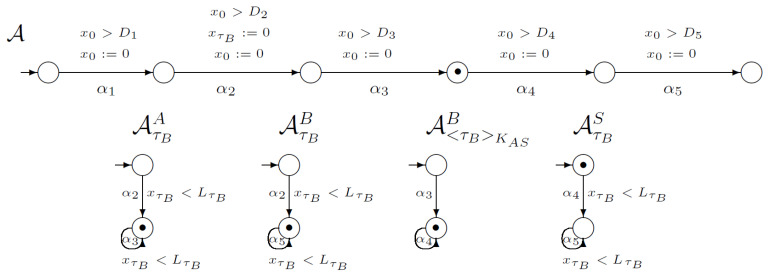
The third step of the WooLamPi protocol.

Now, the third step of the WLP protocol can be executed. Considering Figure 4, we can see that the third transition in automaton *A* is synchronised with the loop in automaton $A_{\tau_{B}}^{A}$ because *A* needs timestamp $\tau_{B}$ for the third step execution. Furthermore, automaton $A_{<\tau_{B}{>}_{K_{AS}}}^{A}$ is synchronised with automaton *A*. During this step, user *B* possesses knowledge about ciphertext $<\tau_{B}{>}_{K_{AS}}$.

**Figure 5:**
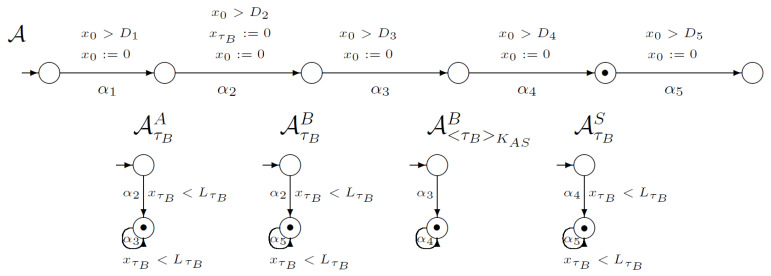
The fourth step of the WooLamPi protocol.

Figure 5 shows that in the fourth step user *B* needs ciphertext $<\tau_{B}{>}_{K_{AS}}$ for executing this step. Additionally, the server *S* possesses a ticket $\tau_{B}$ after decryption of ciphertext $<\tau_{B}{>}_{K_{AS}}$. The four transition in automaton *A* is synchronised with the loop in automaton $A_{<\tau_{B}{>}_{K_{AS}}}^{A}$ and with the automaton $A_{\tau_{B}}^{S}$.

**Figure 6:**
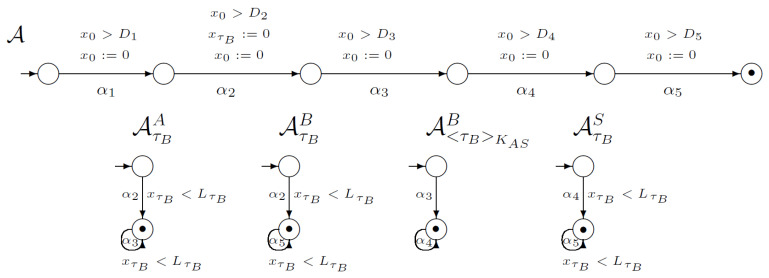
The fifth step of the WooLamPi protocol.

In the last step (Figure 6), the server uses knowledge about a ticked $\tau_{B}$ and the user *B* gets its ticket again (loop in automaton $A_{\tau_{B}}^{B}$).

The method of automatic generation of automata and considered space of the users is given in [12].

The networks of synchronised timed automata for the SNEP protocol’s one honest execution will be constructed similarly. We will consider these networks for both our proposed versions of this protocol.

**Figure 7:**
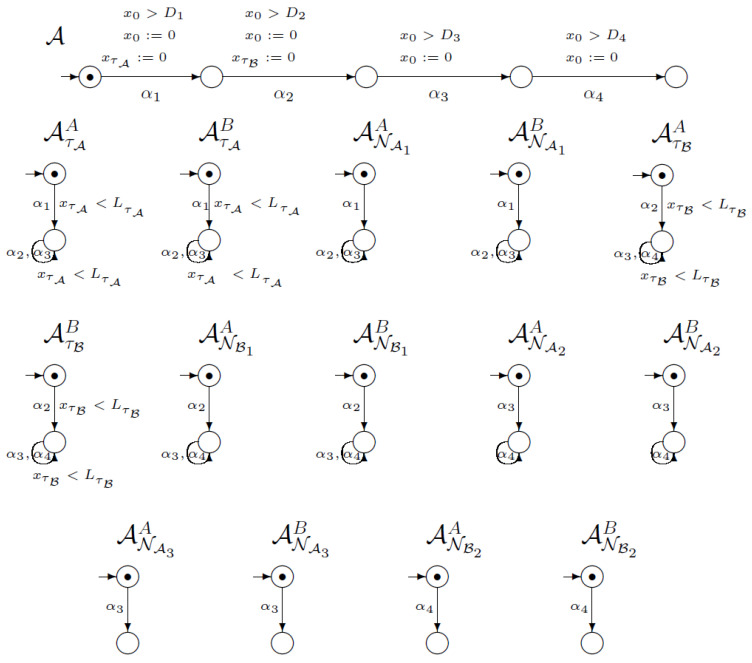
Network of synchronised timed automata for SNEP protocol v1.

Figure 7 shows a part of the network of synchronised timed automata for the first version of the SNEP protocol. This network consists of 22 execution automata and 67 knowledge automata. In the picture we placed one execution automaton and 14 knowledge automata. The execution automaton models execution of four steps of SNEP protocol, including time conditions. Note that on the first ($\alpha_{1}$) transition the clock for timestamp $\tau_{A}$ is reset. In addition, on the second ($\alpha_{2}$) transition, the clock for timestamp $\tau_{B}$ is reset.

The knowledge automaton models the changes in users knowledge about cryptographic objects. For example, the first knowledge automaton $A_{\tau_{A}}^{A}$ models the process of acquisition and use of timestamp $\tau_{A}$ by user *A*. On the first transition ($\alpha_{1}$) user *A* generates timestamp $\tau_{A}$, so the automaton will change its state if the imposed time condition is met. In addition, on the second ($\alpha_{2}$) and third ($\alpha_{3}$) transitions, the loops are defined.

**Figure 8:**
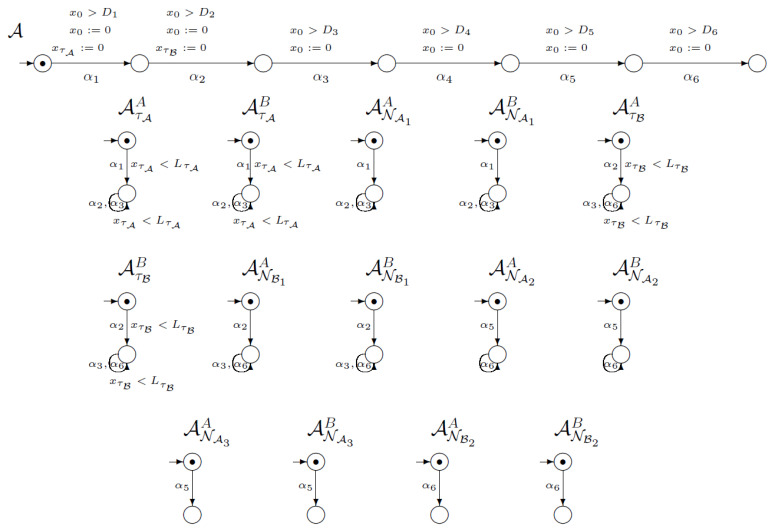
Network of synchronised timed automata for SNEP protocol v2.

Figure 8 shows the network of synchronised timed automata for the second version of the SNEP protocol. This network consists of 22 execution automata and 58 knowledge automata. In the picture, we placed one execution automaton and 14 knowledge automata. In this case, the execution automaton models execution of six steps of the SNEP protocol, including time conditions. The clocks for the timestamps are reset on the same transitions as in Figure 7.

## 5. Reachability Analysis

In this section we formally define the reachability problem for security protocols modelled as a network of timed automata and we present a solution to the problem which uses SMT-solvers and SAT-solvers. We begin with defining the problem and then we describe our solution.

The transition system (timed model) C(A) of a timed automaton A usually has infinitely many states and infinitely many labels. However, the reachability problem of C(A) can be reduced to the reachability problem for a transition system with finitely many states and finitely many labels.

Let $c_{max}$ be the largest constant *c* such that some clock *x* is compared with *c* in some constraint appearing in a guard of A. By $\hat{C}\left(A\right)$ we denote the transition system for a timed automaton which differs from C(A) in the set of labels only: As the set of labels of $\hat{C}\left(A\right)$ we take the set $A\cup [0,c_{max}+1]$.

The region equivalence (the equivalence relation ≃) is defined over the set of all clock valuations for *X*. For two clock valuations *v* and $v^{\prime }$ in ${I\;R}^{|X|}$, we say that $v≃v^{\prime }$ iff for each 0⩽j<n, where *n* is a number of clocks, either $v\left(x_{j}\right)>c_{j}^{max}$ and $v^{\prime }\left(x_{j}\right)>c_{j}^{max}$ or $v\left(x\right)⩽c_{j}^{max}$ and $v^{\prime }\left(x\right)⩽c_{j}^{max}$ and $v\left(x\right)=v^{\prime }\left(x\right)$.

It is a well-known fact, that the relation ≃ is an equivalence relation, which gives rise to the construction of a finite abstract model.

The reachability problem for a network of the timed automata modelled by timed model $\hat{C}\left(A\right)$ is the question of whether for a given set of target locations, a state with a target location is reachable from some initial state. We assume that the set of target locations is described by a propositional formula expressing some property. To check the reachability of a state satisfying the property by the BMC method, first, the transition relation of the model is unfolded iteratively to some depth *k* and encoded as a propositional formula (for SAT-based method) or a quantifier-free first order formula (or SMT-based method) of state variables. Next, the property is translated into a propositional/a quantifier-free first-order formula of the state variables and satisfiability of the conjunction of the two above formulae is checked by a SAT-solver or by an SMT-solver.

If the conjunction, denoted in the algorithm by $\beta_{k}$ is satisfiable, one may conclude that a path to a target location was found. Otherwise, the value of *k* is incremented by 2, as time transitions do not change the global locations (Algorithm 1). The parameter *n* stands for the number of steps of a given protocol.
**Algorithm 1** The standard BMC algorithm BMC for testing reachability1:**procedure**REACHABILITY2:    k:=03:    **loop**4:        $result:=checkSAT(\beta_{k})$5:        **if** result = SATISFIABLE **then**6:           **return** REACHABLE7:        **else if** result = UNKNOWN **then**8:           **return** UNKNOWN9:        **end if**10:        k:=k+211:        **if** *k* > 4 · *n* **then**12:           **return** UNREACHABLE13:        **end if**14:    **end loop**15:**end procedure**

The presented SAT and SMT encoding of the reachability problem for a network of timed automata is based on the SAT encoding presented in [40]. However, we extended the encoding using actions and we also defined a SMT-based encoding.

Let $\hat{C}\left(A\right)$ be a model. To formulate and solve the reachability problem for NTA, we have to define the unfolding of the transition relation to the depth k∈IN. For that purpose, we define a *k*-path to be a finite prefix of a path. Note that arbitrary state q=(l,v) is reachable in $\hat{C}\left(A\right)$ iff it is reachable on a *k*-path, for some k≥0.

We define the formula $path_{k}\left({\bar{w}}_{0},\ldots ,{\bar{w}}_{k}\right)$ which symbolically encodes all the *k*-paths starting at the initial state of $\hat{C}\left(A\right)$. The definition of the formula $path_{k}({\bar{w}}_{0},$$\ldots ,{\bar{w}}_{k})$ assumes that each concrete state q∈Q of $\hat{C}\left(A\right)$ can be represented by a valuation of a symbolic state $\bar{w}=\left(\left(w_{1},v_{1}\right),\ldots ,\left(w_{n},v_{n}\right)\right)$ that consists of symbolic local states. Each symbolic local state is a pair $\left(w_{j},v_{j}\right)$ of individual variables ranging over the natural numbers that consists of a location of the automaton *j* and a clock valuation. Similarly, each action can be represented by a valuation of a symbolic action $\bar{a}$ that is a vector of the individual variables ranging over natural numbers.

In the case of SAT encoding, we use vectors (of the proper length) of propositional variables.

Let $\bar{w}$ and ${\bar{w}}^{\prime }$ be two symbolic states, $\bar{a}$ a symbolic action, and d a symbolic non-negative real number.

We assume definitions of the following quantifier-free first-order formulae: $I_{q}\left(\bar{w}\right)$ encodes the state *q* of the model $\hat{C}\left(A\right)$, $T_{Act}\left(w,\bar{a},w^{\prime }\right)$ encodes an action transition, and $T_{\tau }\left(w,d,w^{\prime }\right)$ encodes a time transition in $\hat{C}\left(A\right)$.

Now for each even k∈IN we can define the formula $path_{k}\left({\bar{w}}_{0},\ldots ,{\bar{w}}_{k}\right)$ as:

$$\begin{array}{c} \underset{q\in s{}^{0}}{⋁}I_{q}\left({\bar{w}}_{0}\right)\wedge \overset{k-2}{\underset{\begin{array}{c} j=0\\ j\;\mathrm{mod}\;2=0 \end{array}}{⋀}}\left(T_{\tau }\left({\bar{w}}_{j},d,{\bar{w}}_{j+1}\right)\wedge T_{Act}\left({\bar{w}}_{j+1},{\bar{a}}_{j},{\bar{w}}_{j+2}\right)\right). \end{array}$$

Using the above formula and a quantifier-free first-order formula $reach(\bar{w})$, which encodes the set of states satisfying a given property, we try to establish whether a state that satisfies $reach(\bar{w})$ is reachable. We do this by checking the satisfiability of the following formula: $\psi_{k}=path_{k}\left({\bar{w}}_{0},\ldots ,{\bar{w}}_{k}\right)\wedge {⋁}_{j=0}^{k}reach\left({\bar{w}}_{j}\right)$. The method described relies on the following theorem.

**Theorem** **1.** *Let $\hat{C}\left(A\right)$ be a model and q be a concrete state. Then for every k∈IN, q is reachable in $\hat{C}\left(A\right)$ on a path of length k if, and only if, the formula $\psi_{k}$ is satisfiable.*

The proof by induction on *k* is straightforward and is presented in [40].

We terminate the unfolding of the transition relation if either the formula $\psi_{k}$ is satisfiable or it is impossible for a given SMT-solver to check satisfiability of the formula in question. We could also terminate if the value of *k* is equal to the reachability diameter of the system—the minimal number of steps required for reaching all the reachable states. Unfortunately, for many systems the diameter cannot be calculated and the estimates are too rough. It makes BMC incomplete in practice.

## 6. Experiments

For research, we used our tool described in [25,26,29,31,40,41,42]. Thanks to it, we generated protocol executions and the network of synchronised timed automata. The network models the protocol executions.

The tests were carried out on a computer unit with the Linux Arch operating system, Intel Core i7-3770 processor, and 32 GB RAM. For satisfiability checking, we used the Yices SAT-solver and the Yices SMT-solver in version 2.6.2 [43].

The network of synchronised timed automata for the WLP protocol consisted of 22 executions automata and 21 knowledge automata. The first two execution automata model honest executions. In those executions, only honest users appears (A, B, and server S). The first automaton represents the execution initiated by A (communication between A, B, and S). The second automaton model the execution initiated by B (communication between B, A, and S). The remaining automata model either executions with an intruder who acts as themself or executions in which the intruder impersonates one of the honest users. In automata 3–12, the intruder either acts as themself or impersonates A, and in automata 13–22, they either act as themself or impersonates B. We have assumed that the intruder could not impersonate a trusted S server.

The network of synchronised timed automata for the SNEP protocol (both versions) consists of 22 executions automata. For the four-step version of the SNEP protocol, the network consists of 47 knowledge automata and 49 knowledge automata for the six-step version. Similarly to the WLP protocol, the first two knowledge automata models executions only with the honest users (A, B). The first automaton represents the execution initiated by A (communication between A and B), while the second automaton models the execution initiated by B (communication between B and A). The remaining automata model either executions with the intruder who acts as themself or executions in which the intruder impersonated one of the honest users. The automata with numbers 3 and 11 model executions with the intruder who acts as themself and appears on the position of the user A. Automata 4–10 and 12 model executions in which the intruder impersonates user A. The automata with numbers 13 and 21 model executions with the intruder who acts as themself and appears on the position of user B. Automata 14–20 and 22 model executions in which the intruder impersonates user B.

Each path in the network of timed automata in which the intruder comes into possession of confidential data is interpreted as a full attack. Paths in which they only stand in the middle of communication impersonating individual users will be interpreted as MiTM behaviour. This behaviour is also undesirable. The mere detection of the presence of the eavesdropping party is important from a security point of view. Time parameters will help us track and, in the long run, limit the possibilities of the intruder.

These studies were related to checking the reachability of states that model protocols executions, including time parameters. For the WLP protocol, we set three different values of delays and two lifetimes values. For the SNEP protocol (both versions), we set five different values of delays and two lifetimes values.

We present the obtained results for the SAT-based and the SMT-based methods in Table 3. We tested the reachability for the last location in each execution automata.

### 6.1. WLP Protocol

**Figure 9:**
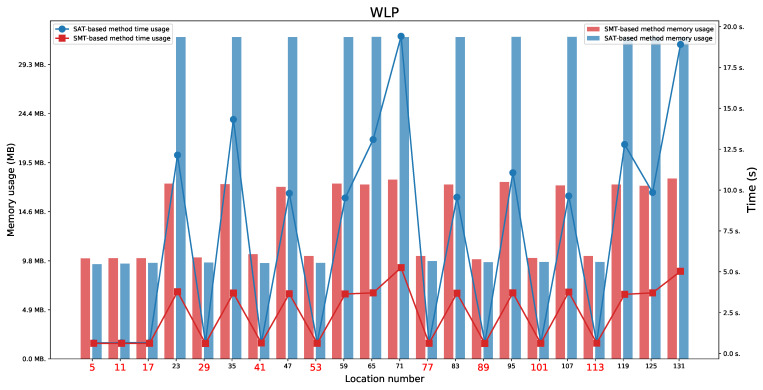
The WLP: Time and memory usage for checking the last locations’ reachability in each execution automaton for both lifetime values equal to 8. The reachable states are marked in red.

**Figure 10:**
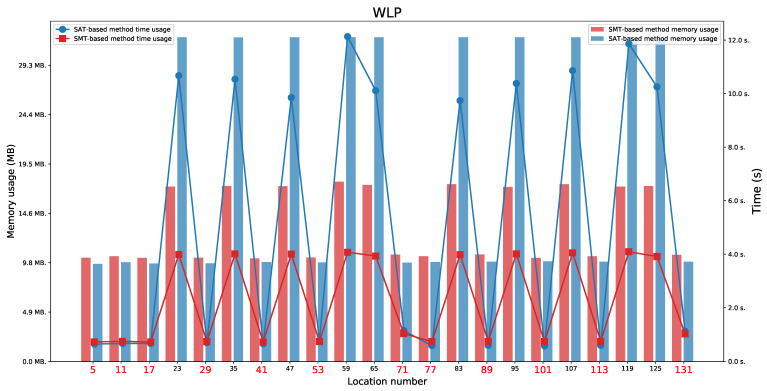
The WLP: Time and memory usage for checking the last locations’ reachability in each execution automaton for both lifetime values equal to 10. The reachable states are marked in red.

For the WLP protocol (Figure 9 and Figure 10), we found two paths indicating a Man in the Middle behaviour (not a full attack). It means that we found the paths on which locations 71 or 131 were reachable. We also observed the availability of two paths that represent an honest execution (the paths ending in one of the locations 5 or 11) and 10 paths representing execution with the intruder (the paths ending in one of the locations 17, 29, 35, 41, 53, 77, 83, 89, 101, or 113). By executions with the intruder, we mean executions in which the intruder acts as a regular user (the paths ending in one of the locations 17, 41, 77, or 101) or tries to impersonate honest users without success (the paths ending in one of the locations 29, 35, 53, 83, 89, or 113).

For the WLP protocol, we observed that values $L_{1}=8$ and $L_{2}=8$ are minimal values which allow to perform both honest executions (these values do not allow a Man in the Middle behaviour). The minimal values that allow to appear a Man in the Middle behaviour for locations 71 and 131 are $L_{1}=10$ and $L_{2}=10$.

The honest executions are possible:

- For location 5 when $L_{1}⩾max\left(D_{1},D_{3}\right)+D_{2}$ and any $L_{2}$,
- For location 11 when $L_{2}⩾max\left(D_{1},D_{3}\right)+D_{2}$ and any $L_{1}$.

The executions which represent a Man in the Middle behaviour are possible:

- For location 71 when $L_{2}⩾max\left(D_{1}+D_{2}+D_{3},2\cdot D_{1}+D_{2}\right)$ and any $L_{1}$,
- For location 131 when $L_{1}⩾max\left(D_{1}+D_{2}+D_{3},2\cdot D_{1}+D_{2}\right)s$ and any $L_{2}$.

### 6.2. Four-Step Version of the SNEP Protocol

**Figure 11:**
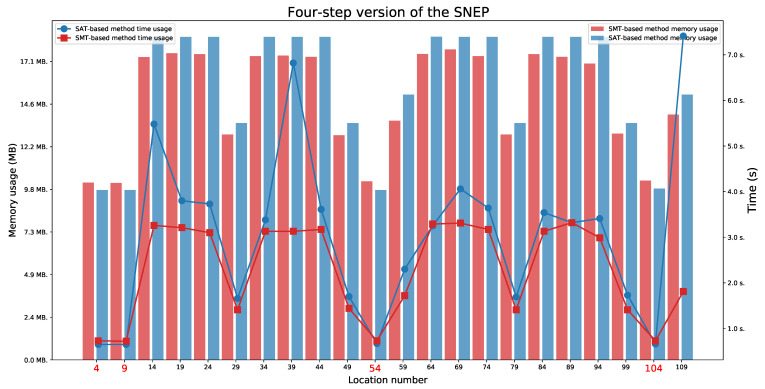
The four-step version of the SNEP: Time and memory usage for checking the last locations’ reachability in each execution automaton for the lifetime value equal to 4. The reachable states are marked in red.

**Figure 12:**
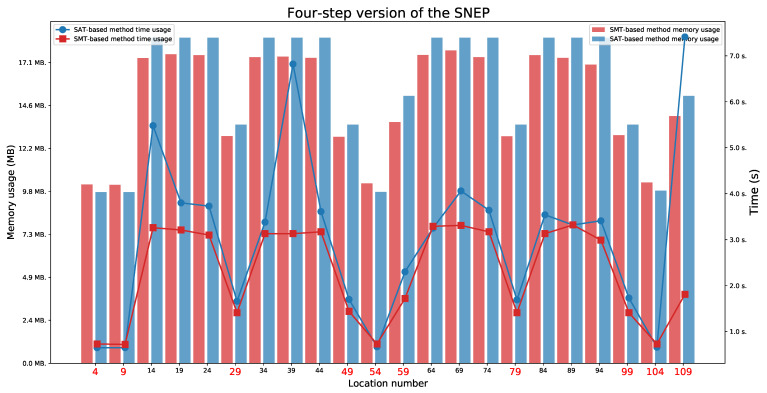
The four-step version of the SNEP: Time and memory usage for checking the last locations’ reachability in each execution automaton for the lifetime value equal to 7. The reachable states are marked in red.

For the four-step version of the SNEP protocol (Figure 11 and Figure 12), we found two paths indicating a Man in the Middle behaviour (the paths ending in one of the locations 49 or 99). The full attack was not found. We also observed the availability of two paths that represent an honest execution (the paths ending in one of the locations 4 or 9) and six paths that represent execution with the intruder (the paths ending in one of the locations 29, 54, 59, 79, 104, or 109).

As before, we set five different values of delays and two lifetimes values for this protocol. We have checked for which time parameter values the protocol executions are feasible. We observed that values $L_{1}=4$ and $L_{2}=4$ are minimal values to allow for both honest executions. These values also do not allow for a Man in the Middle behaviour. The minimal values that allow for a Man in the Middle behaviour for location 49 are $L_{1}=3$ and $L_{2}=7$, and for location 99 are $L_{1}=7$ and $L_{2}=3$.

The honest executions are possible:

- For location 4 when $L_{1}⩾D_{1}$ and $L_{2}⩾D_{1}+D_{2}$,
- For location 9 when $L_{1}⩾D_{1}+D_{2}$ and $L_{2}⩾D_{1}$.

The executions which represent a Man in the Middle behaviour are possible:

- For location 49 when $L_{1}⩾max\left(D_{1},D_{2}\right)$ and $L_{2}⩾D_{1}+D_{2}+D_{3}$,
- For location 99 when $L_{1}⩾D_{1}+D_{2}+D_{3}$ and $L_{2}⩾max\left(D_{1},D_{2}\right)$.

### 6.3. Six-Step Version of the SNEP Protocol

**Figure 13:**
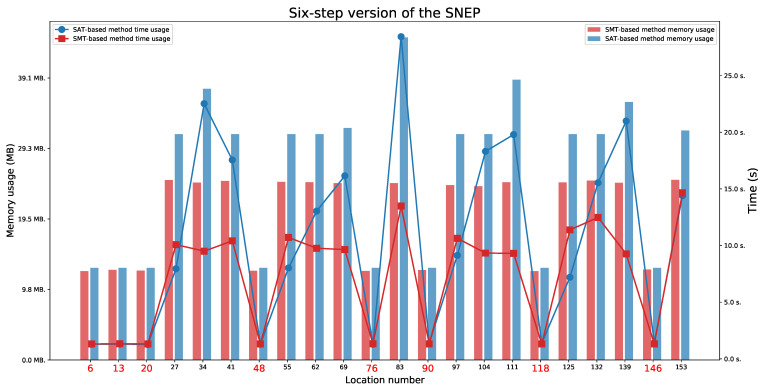
The six-step version of the SNEP: Time and memory usage for checking the last locations’ reachability in each execution automaton for lifetime values equal to 9. The reachable states are marked in red.

**Figure 14:**
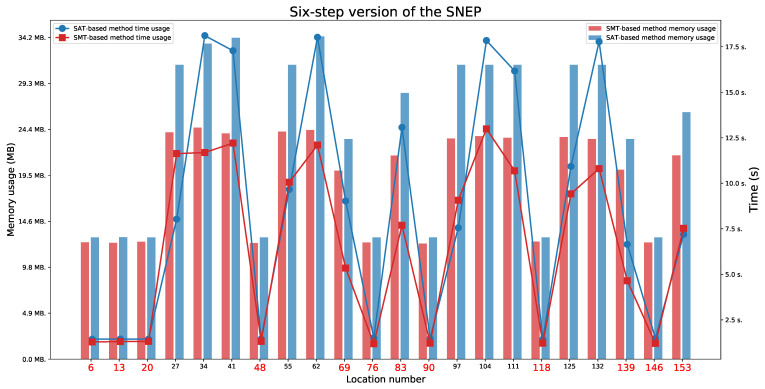
The six-step version of the SNEP: Time and memory usage for checking the last locations’ reachability in each execution automaton for lifetime values equal to 10. The reachable states are marked in red.

For the six-step version of the SNEP protocol (Figure 13 and Figure 14), we found two paths indicating a Man in the Middle behaviour, without full attack (the paths ending in one of the locations 69 or 139), two paths that represent an honest execution (the paths ending in one of the locations 6 or 13), and 10 paths that represent execution with the intruder (the paths ending in one of the locations 20, 34, 48, 76, 83, 90, 104, 118, 146, or 153).

As in the case of the four-step version of the SNEP protocol, we checked for which time parameter values the protocol executions are feasible. We observed that values $L_{1}=L_{2}=9$ are minimal values to allow for both honest executions. These values also do not allow for a Man in the Middle behaviour. The minimal values that allow for a Man in the Middle behaviour are $L_{1}=L_{2}=10$ for locations 69 or 139.

We also observed that the honest executions are possible:

- For location 6 when $L_{1}⩾D_{1}$ and $L_{2}⩾D_{2}+D_{4}$,
- For location 13 when $L_{1}⩾D_{2}+D_{4}$ and $L_{2}⩾D_{2}$.

The executions which represent a Man in the Middle behaviour are possible:

- For location 69 when $L_{1}⩾max\left(D_{1},D_{2}\right)$ and $L_{2}⩾D_{1}+D_{2}+D_{3}$,
- For location 139 when $L_{1}⩾D_{1}+D_{2}+D_{3}$ and $L_{2}⩾max\left(D_{1},D_{2}\right)$.

## 7. Conclusions

In this article, we investigated the correctness of security protocols dedicated to keeping sensor devices communication safe. We presented how we can verify important properties of security protocols using formal mathematical modelling. Accurate modelling allows us to verify whether the protocol works at all, i.e., honest executions reach the final states in the network of automata. We can also check if it is susceptible to an attack, i.e., in the network of automata with the intruder, states representing knowledge about sensitive protocol elements—e.g., nonces are available. The presented model provides an extended security analysis where we investigated time properties. We took into account network delays and the lifetime of timestamps used in the protocol. Those parameters allowed us to detect unwanted network behaviour in which the intruder wants to attack the protocol. Even if a full attack is not possible, we could set the values of time parameters so that even its presence is detectable and the interference is prevented.

In our presentation, we used as examples the SNEP protocol developed for sensors devices communication and different versions of the WooLamPi Protocol. We showed protocols’ schemes and their formal models. These models and the investigated security properties were translated into propositional/quantifier-free first-order formulae. Next, we performed our experiments using our dedicated protocols’ verification tools and SAT and SMT solvers. Finally, we showed experimental results. Our analysis has shown that administrators can select time parameters values in such a way to make the protocol secure.

We have presented our method using relatively simple protocols however, new, more complex protocols have been proposed for securing sensor devices communication [44,45]. Now we plan to take into account and investigate more complex systems. For this, we can use our experience gained during the verification of other systems [17,46].

In the case of protocols designed for sensors, a completely different aspect worth analysing is energy consumption [47]. The limitations of sensors due to the size of resources and batteries make it worth knowing how much energy a given attack requires or how much energy the sensor will use to increase the level of communication security. It may be another interesting research direction.

## Figures and Tables

**Table 1:** Basic notations.

Notations	Explanations
$\alpha_{i}^{j}$	the *i*-th step in the *j*-th execution of the protocol
X·Y	a concatenation of messages *X* and *Y*
A,B,S,I	participants of communication
$I_{U}$	user ID
$T_{U}$	the timestamp created by user *U*
$N_{U}$	pseudorandom number (nonce) generated by user *U*
$K_{UX}$	the encryption symmetric key shared between users *U* and *X*
${\langle D\rangle }_{K}$	the message *D* encrypted with key *K*
${\langle D\rangle }_{M(K)}$	the computation of the message authentication code (MAC)
	of message *D*, with MAC key *K*
${\langle D\rangle }_{K,C}$	the message *D* encrypted with key *K* and the counter *C*
${\langle D\rangle }_{M(K)}$	the computation of the message authentication code (MAC)
	of message *D*, with MAC key *K* and the counter *C*

**Table 2:** Additional notations.

Notations	Explanations
τ	the time of sending the message
$\tau_{U}$	the timestamp created by the user *U*
$L_{\tau_{U}}$	the lifetime of the given timestamp
$D_{i}$	the delay for the given step
$A_{M}^{U}$	the knowledge automaton that represent knowledge of the user
	*U* about the element *M*
$x_{0}$	the global clock
$x_{\tau_{U}}$	the clock for timestamp created by the user *U*

**Table 3:** Experimental results for SAT- and SMT-based methods and protocols (reachability of the last location in each automaton).

Protocol	Yices-SAT		Yices-SMT	
	**Time (s)**	**Memory (kB)**	**Time (s)**	**Memory (kB)**
Four-step version of the SNEP	151.79	21,516	129.05	18,604
Six-step version of the SNEP	418.62	45,748	307.16	25,544
WLP	271.7	32,840	102.86	18,384

## Data Availability

https://cloud.icis.pcz.pl/s/werzTT3tRPmEJQz, accessed on 27 April 2021.
